# Methods of breaking physical dormancy in seeds of the invasive weed* Mimosa pudica* (Fabaceae) and a comparison with 36 other species in the genus

**DOI:** 10.7717/peerj.13567

**Published:** 2022-06-08

**Authors:** Li Tang, Carol Baskin, Jerry Baskin, Kai Luo, Xiaohui Yu, Wei Huang, Rui Zhang, Yinhua Chen

**Affiliations:** 1 College of Tropical Crops, Hainan University, Haikou, China; 2 Department of Plant and Soil Sciences, University of Kentucky, Lexington, KY, USA; 3 Department of Biology, University of Kentucky, Lexington, KY, USA; 4 Archives, Teaching Auxiliary Institutions, Hainan University, Haikou, China

**Keywords:** Dormancy-breaking methods, *Mimosa*, Physical dormancy, Scarification, Water gap

## Abstract

The herbaceous perennial legume *Mimosa pudica* is an invasive weed in many tropical and subtropical regions and a serious problem for farmers since it is difficult to eliminate from crop field by hand. Moreover, it has water impermeable seeds, *i.e.*, physical dormancy (PY), which could persist in the soil seed bank for a long period of time, thus making it a big challenge to control. The aims of this study were to test the effect of various laboratory methods on breaking PY in seeds of *M. pudica*, to identify the site(s) of water entry into seeds of *M. pudica* and compare results of dormancy-breaking methods for seeds of *M. pudica* with those of 36 other species of *Mimosa* reported in the literature. Mechanical scarification, wet heat and cycles of wet heat and ice water effectively broke PY in seeds of *M. pudica*. Following wet heat at 80 °C for 10 min, water uptake was *via* the hilar region but not the pleurogram; small cracks made in the pleurogram by this treatment were not deep enough for water to enter the seed. Neither tolerance to summer temperatures nor PY is likely to be the cause of invasiveness of this species, since seeds of rare and endemic species of* Mimosa* also tolerate summer temperatures and have PY.

## Introduction

Physical dormancy (PY) is caused by (a) water-impermeable layer(s) of palisade cells in the seed/fruit coat that are impregnated with various water-repellent substances (see (Baskin & Baskin, 2014) and references cited therein). Water-impermeability develops as seeds undergo maturation drying (Baskin & Baskin, 2014; Liu et al., 2016). PY is known to occur in 18 families of angiosperms (Baskin & Baskin, 2014), including Fabaceae, the family to which our study species belongs. Seeds/fruits with PY have a structure on the coat that is dislodged in response to environmental signals, thereby creating an opening (water gap) in the seed/fruit coat that allows water to enter the seed. Relevant to the research reported in this study, various structures on the seeds of Fabaceae may serve as water gaps, including the lens, micropyle, hilar slit (Gama-Arachchige et al., 2013) and pleurogram (Rodrigues-Junior et al., 2021).

The pleurogram is an area on both sides (“faces”) of the bilaterally symmetrical seeds of some Fabaceae (subfamilies Mimosoideae and Caesalpinioideae but not Papilionoideae). In the Mimosoideae, the pleurogram is delimited by a U-shaped groove (or line) that usually is open toward the hilar end of the seed in the Mimosoideae, whereas in the Caesalpinioideae the groove usually is closed (Corner, 1976; Manning & Van Staden, 1987; Rodrigues-Junior et al., 2021). Further, the pleurogram is an area of structural weakness and recently was shown to act as a water gap in some species of *Senna* (Fabaceae, subfamily Caesalpinioideae) (Rodrigues-Junior et al., 2019; Rodrigues-Junior et al., 2021). It should be noted that various authors have defined the pleurogram as only the groove or line with no function. For example, Kopooshian & Isely (1966) wrote that “…the pleurogram is a horseshoe-shaped line, a groove in the seed coat, following the curve of the flat surface of the seed and usually open at the hilar end [in Fabaceae subfamily Mimosoideae]”.

*Mimosa* is one of the largest genera in the Fabaceae, comprising ca. 530 species worldwide ranging from small herbs to trees (Mabberley, 2017). *Mimosa pudica* (Fabaceae, subfamily Mimosoideae) is native to tropical America and is an invasive species in Tanzania, southern and southeastern Asia, many Pacific Islands, the Northern Territory of Australia (Holm et al., 1977; Joseph, George & Mohan, 2013) and southern China (Li, 2018). Previous studies showed that seeds of *M. pudica* have PY (Nava-Rodriguez, 1974; Chauhan & Johnson, 2009; Niroula, Parajuli & Jha, 2009; Jayasuriya et al., 2013) and that mechanical scarification, chemical scarification (immersion in sulphuric acid) and dry heat could break dormancy. However, the mechanics of dormancy break of *M. pudica* has not been studied, and studies on the function of pleurogram in seeds of *Mimosa* species are rare. da Silva et al. (2017) found that although there are multiple fissures in the pleurogram of *M. caesalpiniifolia* seeds given an atmospheric cold plasma treatment to break dormancy, they were not deep enough to allow water to penetrate the water-impermeable palisade layer of cells. Geisler et al. (2017) showed that imbibition in *M. bimucronata* seeds was *via* the hilar region and that the pleurogram was not a site of water uptake.

Our objectives were to (1) test the effect of various methods known to break PY in Fabaceae on *M. pudica* seeds, (2) identify the site(s) of water entry into seeds of *M. pudica* and (3) compare results of dormancy-breaking methods for seeds of *M. pudica* with those of 36 other species of *Mimosa* reported in the literature. The results will be contribute to a better understanding of seed dormancy mechanism and germination of *M. pudica* and other *Mimosa* species.

## Material and Methods

### Seed collection site and seed size

Mature seeds of *M. pudica* were collected in mid-August 2020 from plants growing on roadsides near the field station of Hainan University, Haikou, Hainan Province, China (20.07°N, 110.33°E, 4 m a.s.l.). Mean annual temperature is 25.8 °C and mean annual rainfall 1798.7 mm. Mean monthly rainfall from May to October is 281.3 mm (wet season), while it is only 18.5 mm for November to April (dry season). These data were purchased from the Hainan Meteorological Service.

Seed width, length and thickness of 20 haphazardly-selected seeds of *M. pudica* were measured using an electronic Vernier caliper (111N-101v-10G; Guanglu, Guilin, China). Four groups of 1,000 mature seeds were weighed with an electronic balance (0.0001 g) (WBA220; Daihan, South Korea) to determine seed mass. Seed features were measured immediately after seed collection.

### Effect of mechanical scarification on imbibition and germination

We tested germination of freshly-collected seeds of *M. pudica* seeds immediately after they were collected and cleaned in the laboratory. Imbibition of water at 25 °C by scarified and intact seeds was compared to confirm (or not) whether the seeds were water impermeable. For the intact and scarified seeds (scarified individually away from the hilar using a razor blade), four replicates each of 25 seeds were used. Each group of seeds was weighed and then placed in 9-cm-diameter Petri dishes on two layers of filter paper moistened with distilled water. After 1, 2, 3, 4, 5, 6, 8, 10, 12 and 25 h, seeds were removed from the Petri dishes, blotted dry, weighed and returned to the dishes. Percentage increase in seed mass was determined as [(*W*_g_–*W*_i_)/*W*_i_] ×100, where *W*_i_ is the initial fresh seed mass and *W*_g_ is the mass after a given period of time (Long et al., 2012).

For both intact and scarified seeds, four replicates each of 25 seeds were placed in Petri dishes on two layers of Whatman No. 1 filter paper moistened with distilled water and incubated for 14 days at 20/10, 25/15, 30/20, 35/25, 40/30 and 40 °C in light (12 h light/12 h dark, hereafter light) or in constant darkness (dishes wrapped with aluminum foil). The light source was white fluorescent tubes, and photon irradiance at seed level was 60 µmol m^−2^ s^−1^ (400–700 nm). Germination in light was checked daily and water added to the dishes if needed. Germination in darkness was determined only at the end of the 14-day test. The criterion for germination was a radicle protruding from the seed for at least one mm.

### Effect of wet heat, dry heat and alternating wet heat and ice water on seed dormancy break

Prior to treatment, seeds were incubated on two layers of water-moistened filter paper at 25 °C for 25 h, and imbibed seeds were discarded. Thus, only water-impermeable seeds (PY) were included in the tests (only a few intact seeds imbibed). For dry heat treatments, four groups of 25 dormant seeds each were heated at 65, 80 and 95 °C in a laboratory drying oven for 1, 10 or 30 min. For wet heat treatments, four groups of 25 dormant seeds each were placed in nylon bags (10  × 15 cm) and exposed to 65, 80 and 95 °C for 1, 10 or 30 min in a water bath (TWS-12; Zhetu Corp., Shanghai, China). In a third series of treatments, dormant seeds were placed in nylon bags and dipped in 95 °C water for 10 s and then transferred to ice water (0 °C) for 2 min (alternating wet heat and ice water). Seeds were subjected to 1, 5, 10 and 30 hot water/cold water cycles.

After treatments, seeds were incubated in light at 25/15 °C for 14 days. At the end of the germination tests, nongerminated seeds were cut open to determine whether they were viable or nonviable (Baskin & Baskin, 2014). Nongerminated seeds with a firm, white embryo were recorded as viable and those with a soft, grey embryo as nonviable (Li et al., 2017). Percentages of germinated, viable nongerminated and nonviable seeds were calculated.

### Site of water uptake by seeds

Seeds that had received a wet heat treatment at 80 °C for 10 min to break dormancy were used to locate the site of water absorption. Four replicates of 25 seeds each were subjected to two blocking treatments. Thus, a blocking material (Vaseline) (Yousif, Wang & Hu, 2019; Hu & Wang, 2021) was applied with a toothpick to cover (1) the hilar region, including the hilum, lens and micropyle, and (2) the pleurogram. The controls were whole seeds blocked and whole seeds not blocked. Seeds were placed in Petri dishes on two layers of filter paper moistened with distilled water incubated and 14 days at 25/15 °C in light, and germination was monitored daily. The effectiveness of Vaseline as a blocking material previously had been shown by testing it on mechanically-scarified *vs.* intact seeds.

### SEM of seeds

Ten intact seeds and ten seeds that had received wet heat at 80 °C for 10 min to break dormancy were washed separately with 0.1 mol/L PB (phosphate buffer, pH 7.4) three times for 15 min. Then, they were transferred to 1% OsO_4_ in 0.1 mol/L PB for 1–2 h at room temperature. Next, seeds were treated with 0.1 mol/L PB three times for 15 min each, after which they were dehydrated in an ethanol-dehydration series (30% → 50% →70% → 80% → 90% → 95% →100%), remaining in each concentration for 15 min. The dehydrated seeds were placed in isoamyl acetate for 15 min, after which they were put in a Critical Point Dryer (Quorum K850, England) for 3 h. Seeds were attached to metallic stubs using carbon stickers and sputter-coated with gold for 30 s, and then they were observed using a scanning electron microscope (SEM) (HITACHI Regulus 8100, Japan) at an acceleration voltage of 30 kV (Chen et al., 2019; Montaño Arias et al., 2022).

### Dormancy-breaking methods for physically dormant seeds of *Mimosa*

Research papers on effects of various laboratory treatments on dormancy-break in seeds of *Mimosa* were searched for on Google Scholar (https://scholar.google.com) using the keywords “*Mimosa*”, “seed”, and “dormancy break”. Nomenclature follows The Plant List (http://www.theplantlist.org/). The section for each species of *Mimosa* follows Barneby (1991).

### Statistical analysis

Mann–Whitney U Test were used to analyze the percentage increase in fresh mass of intact and scarified seeds after 25 h of imbibition. Kruskal-Wallis analysis of variance was used to test the effects of dormancy break methods (mechanical scarification, dry heat, wet heat, alternating wet heat and ice water cycles), temperatures and blocking with Vaseline on seed germination of *M. pudica*. All analyses were performed with SPSS Version 20.0 (SPSS Inc., Chicago, IL, USA).

## Results

### Seed size

Mature seeds of *M. pudica* were yellow-brown and ovoid, and 1000 seed weight was 5.74 ± 0.06 g (mean ± s.e). Seed length, width and thickness were 2.86 ± 0.08 mm, 2.69 ± 0.14 mm and 1.33 ± 0.05 mm, respectively.

### Effect of mechanical scarification on imbibition and germination

**Figure 1:**
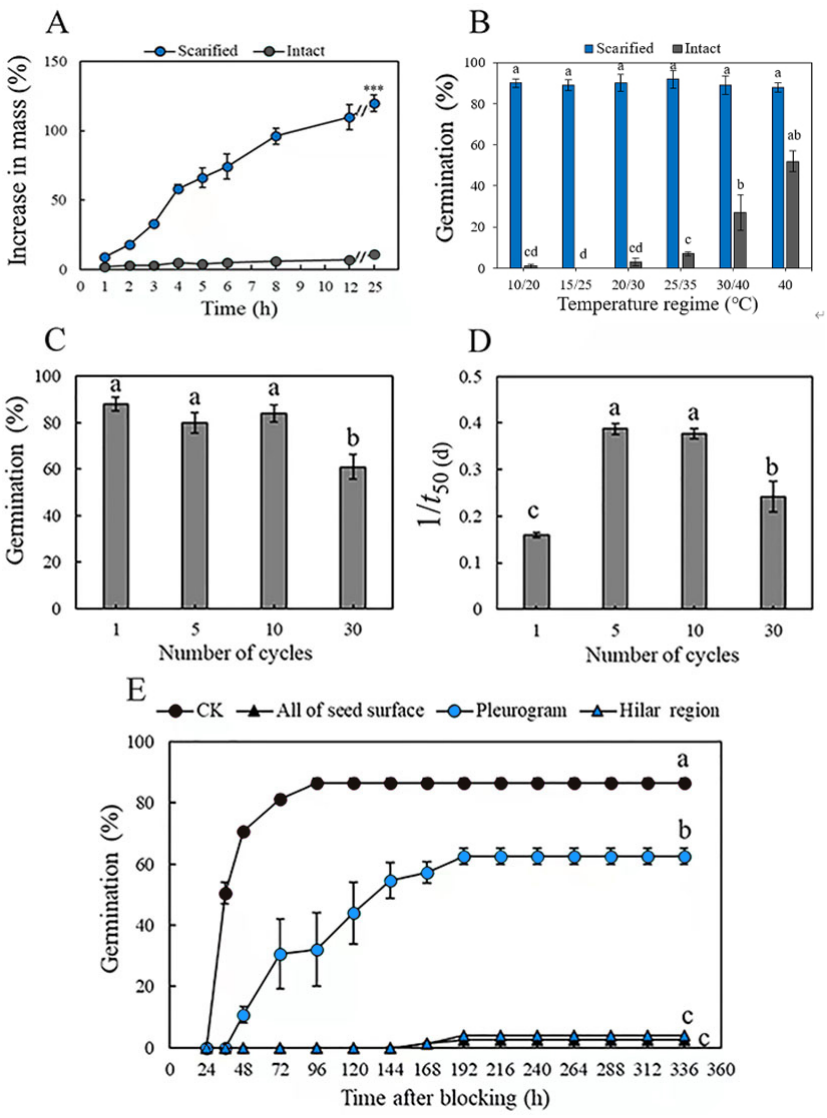
(A) Increase in mass (mean % ± se) of mechanically scarified and intact seeds of *M. pudica* after 25 h, “***” indicate significant (*P*< 0.05) difference in percentage increase in mass at 25 h between the two treatments. (B) Germination percentage of scarified and intact seeds of *M. pudica* at different temperature regimes in light. Different lowercase letters indicate significant (*P*< 0.05) differences among treatments. The same as blow in C, D, E. (C, D) Effects of alternating cycles of wet heat and ice water on germination percentage (C) and germination rate (1/*t*_50(d)_) (D) of intact seeds of *M. pudica* (E) Cumulative germination curves for *M. pudica* seeds with hilar region, pleurogram, all of seed surface or nothing (CK) blocked.

Mass of the scarified seeds increased by 32 ± 3% in 3 h and by 129 ± 2% (fully imbibed) in 25 h, whereas that of intact seeds increased by only 11 ± 3% in 25 h, as a result of only a few seeds (3.0 ± 1.9%) imbibing (Fig. 1A). Mechanical scarification effectively broke dormancy of *M. pudica* seeds*,* and temperature significantly (*P* < 0.05) affected germination percentage of both scarified and intact seeds. Germination of scarified seeds was >89% at all five temperature regimes, while the highest germination of intact seeds was only 27%, at 40/30 °C in light, which was significantly higher than it was for intact seeds at the other four temperature regimes (Fig. 1B).

### Effect of wet heat, dry heat and alternating wet heat and ice water on seed dormancy break

Alternating wet heat and ice water, wet heat and dry heat broke PY in a maximum of 88, 86 and 50% of the seeds, respectively (Table 1, Fig. 1C). For wet heat treatments, germination (1) increased gradually with an increase in exposure time from 1 min to 30 min at 65 °C: 50% for 30 min >46% for 10 min >35% for 1 min, and nonviability was 8% after 1 min, 5% after 10 min and 9% after 30 min; (2) increased and then decreased with an increase in exposure time from 1 min to 30 min at 80 °C: 86% for 10 min >69% for 1 min >39% for 30 min, and nonviability was 12% after 1 min, 9% after 10 min and 56% after 30 min; and (3) decreased with increasing exposure time from 1 min to 30 min at 95 °C: 79% for 1 min >63% for 10 min >47% for 30 min, and nonviability was 15% after 1 min, 35% after 10 min and 46% after 30 min. For dry heat treatments, the highest germination (50%) was obtained for seeds exposed to 95 °C for 10 min. The percentage of nonviable seeds was higher for seeds exposed to wet heat than to dry heat (Table 1). For alternating wet heat and ice water treatments, germination percentages after one, five and ten cycles did not differ significantly, but they (one, five and ten cycles) were significantly higher than those for seeds given 30 cycles. The highest germination (88%) was obtained for seeds after 1 cycle, and treatments with 10 cycles showed the highest germination rate (Figs. 1C, 1D).

**Table 1:** Effects of dry heat and wet heat treatments on germination at 25/15 °C and viability of intact seeds of *M. pudica*. Different lowercase letters indicate significant differences among different temperatures and times under the same treatment (*P* < 0.05). CK is intact seed with no dormancy breaking treatment, *i.e.* control.

Treatment	Temperature (°C)	Time (min)	Germination (%)	Viable (%)	Viable nongerminated (%)	Nonviable (%)
CK	–	–	0	100	100	0
Wet heat	65	1	35 ± 6^d^	92 ± 3^ab^	57 ± 7^a^	8 ± 3^bc^
		10	46 ± 3^cd^	95 ± 2^a^	49 ± 2^a^	5 ± 2^c^
		30	50 ± 6^bcd^	91 ± 4^ab^	41 ± 6^a^	9 ± 4^bc^
	80	1	69 ± 6^abc^	88 ± 2^ab^	19 ± 6^b^	12 ± 2^bc^
		10	86 ± 5^a^	91 ± 4^ab^	5 ± 2^b^	9 ± 4^bc^
		30	39 ± 9^cd^	44 ± 12^c^	5 ± 3^b^	56 ± 12^a^
	95	1	79 ± 3^ab^	85 ± 5^ab^	6 ± 3^b^	15 ± 5^bc^
		10	63 ± 8^abcd^	65 ± 8^bc^	2 ± 2^b^	35 ± 8^ab^
		30	47 ± 6^cd^	54 ± 4^c^	7 ± 3^b^	46 ± 4^a^
Dry heat	65	1	2 ± 1^ef^	100 ± 0	98 ± 1^a^	0 ± 0
		10	8 ± 3^cde^	97 ± 2	89 ± 5^abc^	3 ± 2
		30	6 ± 3^def^	100 ± 0	94 ± 3^ab^	0 ± 0
	80	1	0 ± 0^f^	99 ± 1	99 ± 1^a^	1 ± 1
		10	22 ± 6^bcd^	99 ± 1	77 ± 6^bc^	1 ± 1
		30	25 ± 2^bc^	99 ± 1	74 ± 2^c^	1 ± 1
	95	1	1 ± 1^ef^	97 ± 1	96 ± 2^ab^	3 ± 1
		10	50 ± 8^a^	95 ± 2	45 ± 6^d^	5 ± 2
		30	44 ± 6^ab^	92 ± 4	48 ± 6^d^	8 ± 4

### Site of water uptake by seeds

Seeds that had received a wet heat treatment at 80 °C for 10 min to break dormancy and blocked at the hilar region (hilum, micropyle and lens) and over the whole seed germinated to only 3% and 4%, respectively, after 192 h. However, germination of seeds blocked at the pleurogram and non-blocked on the whole seed surface was 69% and 87%, respectively (Fig. 1E).

### SEM of seeds

**Figure 2:**
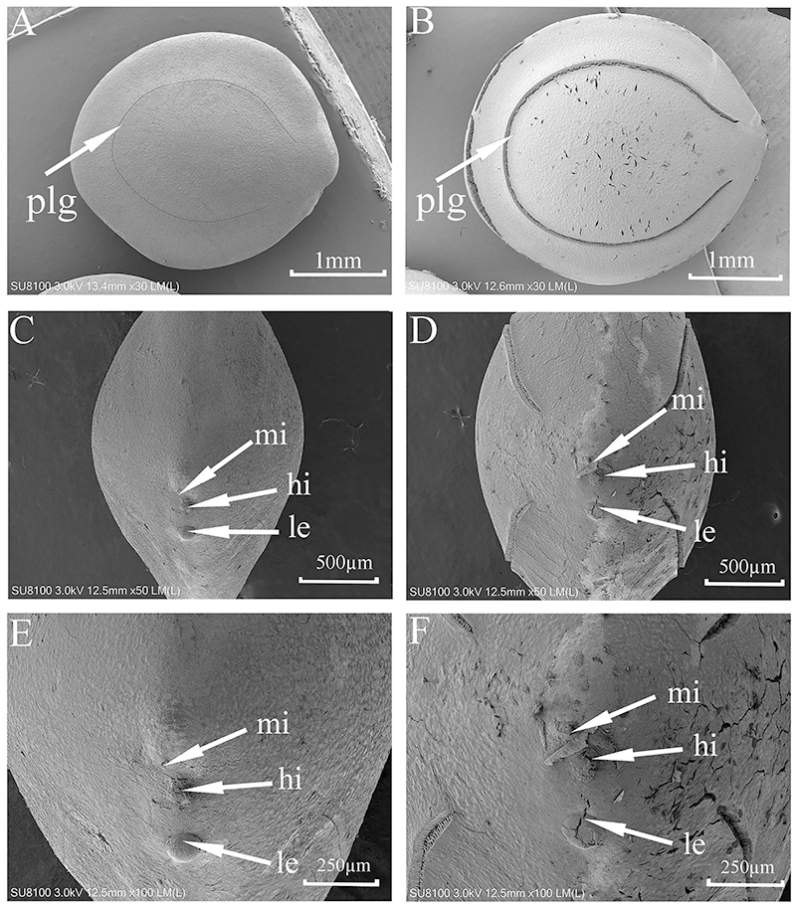
Scanning electron micrographs of seed coat surface of *M. pudica*. (A, C, E) intact seed; (B, D, F) seeds exposed to wet heat at 80 °C for 10 min to break PY; plg, line delineating the outer boundary of the pleurogram; le, lens; hi, hilum; mi, micropyle. Scale bars: 1 mm (A–B), 500 µm (D–F).

SEM micrographs (Fig. 2) showed some cracks on the seed surface after wet heat treatment (80 °C, 10 min), especially in the lens and extrahilar regions. The hilum and micropyle clearly were opened, and there was an obvious slit on the slightly raised lens after wet heat treatment (80 °C, 10 min). There were no obvious cracks in the hilum, lens or extrahilar regions of the intact seeds. Some cracks were present in the pleurogram, and the pleurogram delineation line (groove) was wider on seeds given a wet heat treatment (80 °C, 10 min) but not in intact seeds.

### Dormancy-breaking methods for physically dormant seeds of *Mimosa*

Our literature survey revealed information on laboratory treatments to break PY in seeds of 36 species of *Mimosa*, in addition to *M. pudica*, and these species belonged to four of the five sections of the genus (see Barneby, 1991) (Table 2). Of the various laboratory treatments used to break PY in seeds of the 36 species of *Mimosa*, mechanical scarification, wet heat and chemical scarification (immersion in sulphuric acid) were the most frequently used treatments, but mechanical scarification was the most effective treatment in breaking PY of *Mimosa* seeds (Table 2). High temperatures can break PY in many species of *Mimosa*, but wet heat was more efficient for dormancy-break than dry heat for seeds of *M. tenuiflora* (Araujo & Andrade, 1983), *M. bimucronata* (Geisler et al., 2017), *M. diplotricha* (Nawa, 2014; Aigbokhan, Osazuwa-Peters & Ilubon, 2010), *M. regnellii* (Fowler & Carpanezzi, 1997), *M. dolens* (Leal & Biondi, 2007), *M. caesalpiniifolia* (Leal et al., 2008), *M. strobiliflora* (Biondi & Leal, 2008), *M. pudica* (Chauhan & Johnson, 2009), *M. setosa* (Sperandio, Lopes & Matheus, 2013) and *M. flocculosa* (Ribeiro et al., 2020).

**Table 2:** Effect of dormancy-breaking treatments on, and geographical distribution/ecological status of, various taxa of *Mimosa*.

Species	Section	Treatment	Effect of dormancy- breaking treatment	Imbibition tested (yes)/ just mentioned seeds have PY (M)	Native distribution and ecological status	References
*M. aculeaticarpa*	Batocaulon	mechanical scarification	+	M	Mexico, SE USA, endemic	Montaño Arias et al. (2015)
*M. adenantheroides*	Batocaulon	mechanical scarification	+	M	Central and Southern Mexico, Honduras	Camargo-Ricalde, Dhillion & Garcıa-Garcıa (2004)
*M. bimucronata*	Batocaulon	wet heat	++	M	Bolivia to Brazil and NE Argentina, invasive weed	Giasson et al. (2019)
		H_2_SO_4_ scarification	+			
		wet heat	++*	M		Nogueira (1996)
		H_2_SO_4_ scarification	+			
		wet heat	++	M		Geisler et al. (2017)
		dry heat	+			
		cold stratification	–	Not M		Wang et al. (2016)
*M. borealis*	Batocaulon	mechanical scarification	+	M	W central and central USA to NE Mexico	Orozco-Almanza et al. (2003)
*M. caesalpiniifolia*	Batocaulon	cold stratification	–	M	Brazil	Dos Santos, De Oliveira & De Oliveira (2013)
		H_2_SO_4_ scarification	+			
		wet heat	+			
		mechanical scarification	++			
		sodium hypochlorite	–			
		wet heat	+	M		Leal et al. (2008)
		atmospheric pressure cold plasma	+	yes		da Silva et al. (2017)
*M. calcicola*	Batocaulon	mechanical scarification	+	M	Mexico	Camargo-Ricalde, Dhillion & Garcıa-Garcıa (2004)
*M. calodendron*	Calothamnos	mechanical scarification	+	M	SE Brazil, endemic	Dayrell et al. (2015)
*M. chaetocarpa*	Mimosa	dry stored	++	M	Central Mexico	Moreno-Casasola, Grime & Martínez (1994)
		alternatively wet and dried	+			
*M. depauperata*	Batocaulon	mechanical scarification	+	M	Mexico	Orozco-Almanza et al. (2003)
*M. diplotricha*	Batocaulon	H_2_SO_4_ scarification	+	M	Tropical and subtropical America, invasive weed	Nawa (2014)
		wet heat	+			
		infrared radiation	–			
		mechanical scarification	++			
		burn (fire)	–	Not M		Aigbokhan, Osazuwa-Peters & Ilubon (2010)
*M. dolens*	Mimosa	wet heat	+	M	E Bolivia to Brazil and NE Argentina	Leal & Biondi (2007)
*M. echinocaula*	Batocaulon	dry heat	–	M	W central Brazil	Zirondi, Silveira & Fidelis (2019)
*M. flocculosa*	Calothamnos	H_2_SO_4_ scarification	+	M	W central and S Brazil to Paraguay	Ribeiro et al. (2020)
		wet heat	++			
		H_2_SO_4_ scarification	+	M		Shibata et al. (2017)
*M. foliolosa*	Habbasia	H_2_SO_4_ scarification	+	M	Brazil	Silveira & Fernandes (2006)
		mechanical scarification	++			
		dry heat	–	M		Zirondi, Silveira & Fidelis (2019)
		smoke	–			
		dry heat and smoke	–			
		mechanical scarification	+	M		Silva & Fernandes (2014)
		stored at room temperature	–			
*M. himalayana* (*M. rubicaulis*)^a^	Batocaulon	Stored dry in tin boxes at room temperature for 1 year	+	M	Indian subcontinent	Rana, Nautiyal & Bisht (2007)
		mechanical scarification (puncturing) of fresh seeds	–			
		mechanical scarification (puncturing) after 1 year of dry storage	+			
		mechanical scarification of fresh seeds (rubbing with sand paper)	+			
		mechanical scarification (rubbing with sand paper) after 1 year of dry storage	++*			
		H_2_SO_4_ scarification of fresh seeds	+			
		H_2_SO_4_ scarification after 1 year of dry storage	++*			
		wet heat of fresh seeds	–			
		wet heat after 1 year of dry storage	+			
*M. invisa*	Batocaulon	H_2_SO_4_ scarification	++	M	Colombia to Venezuelaand Paraguay, invasive weed	Chauhan & Johnson (2008)
		dry heat	+			
*M. lacerata*	Batocaulon	mechanical scarification	+	M	Mexico	Camargo-Ricalde, Dhillion & Garcıa-Garcıa (2004)
				M		Orozco-Almanza et al. (2003)
*M. laticifera*	Batocaulon	dry heat	–	Not M	C and E Brazil	Rizzini (1976)
		mechanical scarification	+			
*M. leiocephala*	Habbasia	dry heat	+	M	C Brazil	Zirondi, Silveira & Fidelis (2019)
		smoke	–			
		dry heat and smoke	++			
*M. luisana*	Batocaulon	mechanical scarification	+	M	Mexico	Montaño Arias et al. (2015)
		mechanical scarification	++	M		Montaño Arias et al. (2021)
		ingested by goat	+	M		Giordani (2008)
*M. monancistra*	Batocaulon	wet heat	–	M	Mexico	González-Castañeda et al. (2004)
		H_2_SO_4_ scarification	++			
		mechanical scarification	+			
*M. multipinna*	Habbasia	mechanical scarification	+	Not M	Brazil	Rizzini (1976)
*M. ophthalmocentra*	Batocaulon	H_2_SO _4_scarification	+	M	Cand E Brazil	Brito et al. (2014)
		20% caustic soda	–			
		commercial bleach	–			
*M. pigra*	Habbasia	mechanical scarification	++	M	Tropical and subtropical America, invasive weed	Marambe et al. (2004)
		H_2_SO_4_ scarification	+	Not M		Dillon & Forcella (1985)
		mechanical scarification	+	yes		Jayasuriya et al. (2013)
*M. pudica*	Mimosa	dry heat	+	M	Native to tropical America, cultivated around the world, invasive weed	Chauhan & Johnson (2009)
		wet heat	+			
		H_2_SO_4_ scarification	+			
		mechanical scarification	++			
		cold stratification	–	Not-M		Wang et al. (2016)
		mechanical scarification	+	yes		Jayasuriya et al. (2013)
		mechanical scarification	++*	M		Niroula, Parajuli & Jha (2009)
		H_2_SO_4_ scarification				
		7-year-old seeds	–			
*M. pilulifera*	Calothamnos	H_2_SO_4_ scarification	+	M	SE and S Brazil to NE Argentina	Fowler & Carpanezzi (1999)
		wet heat	++			
*M. polyantha*	Batocaulon	mechanical scarification	+	M	Mexico, Colombia	Camargo-Ricalde, Dhillion & Garcıa-Garcıa (2004)
*M. pteridifolia*	Batocaulon	dry heat	–	M	Brazil	Zirondi, Silveira & Fidelis (2019)
		smoke	–			
		dry heat and smoke	+			
*M. regnellii*	Mimosa	H_2_SO_4_ scarification	+	Not M	SE and S Brazil to NE Argentina, rare	Fowler & Carpanezzi (1997)
		wet heat	++			
*M. scabrella*	Calothamnos	wet heat	+	M	SE to S Brazil to NE Argentina, invasive weed	Bianchetti (1981)
		H_2_SO_4_ scarification	++			
		wet heat	+	M		da Rosa et al. (2012)
		mechanical scarification	++*			
*M. setosa*	Habbasia	H_2_SO_4_ scarification	+	M	Brazil to Paraguay	Sperandio, Lopes & Matheus (2013)
		wet heat	++			
		KNO_3_	–			
*M. similis*	Batocaulon	mechanical scarification	+	M	SW. Mexico	Orozco-Almanza et al. (2003)
*M. skinneri var. carajarum*	Mimosa	mechanical scarification	+	M	N Brazil	Zanetti et al. (2020)
*M. somnian*	Habbasia	dry heat	–	M	Mexico to S tropical America to Trinidad, weed	Zirondi, Silveira & Fidelis (2019)
		smoke	+			
		dry heat and smoke	+			
		freezing (liquid nitrogen)	++	M		Salomão (2002)
*M. strigillosa*	Habbasia	cold stratification	–	Not M	S USA to Mexico, W central Brazil to N Argentina	Walker (2006)
		wet heat	–			
		mechanical scarification	+			
		H_2_O_2_	–			
		wet heat/cold stratification	–			
*M. strobiliflora*	Batocaulon	wet heat	+	M	Brazil, threatened species	Biondi & Leal (2008)
*M. tenuiflora*	Habbasia	wet heat	+	Not M	S Mexico to Venezuela, NE Brazil	Araujo & Andrade (1983)
		H_2_SO_4_ scarification	+			
		mechanical scarification	++			
		dry heat	–			
		mechanical scarification	++	M		Camargo-Ricalde & Grether (1998)
		H_2_SO _4_scarification	+			
		heat from fire	–			

**Notes.**

+, germination percentage significantly higher than that of control or if statistical test was not done ≥ 30 % of the control; ++, treatment with the highest germination when multiple treatments were given for the same study; ++*, treatment with the highest germination percentage when multiple treatments were given in different studies; -, no significant increase in germination percentage or < 30 % of control; yes, imbibition of scarified and nonscarified seeds compared; M, authors said that seeds had physical dormancy. Geographical distribution data are from the Royal Botanical Gardens, Kew, Plants of the World online, 6 August 2021. a Fresh and 1-year-old (dry stored) seeds of *M. himalayana* rubbed with sand paper germinated to 98 and 100%, respectively, suggesting that seeds have only PY. However, the lower germination of fresh punctured, wet heat- and acid-treated seeds compared to 1-year-old seeds (Rana, Nautiyal & Bisht, 2007) suggests that seeds have PY+PD.

## Discussion

Most fresh intact seeds of *M. pudica* did not imbibe water, while all mechanically scarified seeds imbibed water rapidly. In addition, 89% or more of scarified seeds germinated across the range of temperatures, indicating that *M. pudica* seeds have PY and not physical (PY) plus physiological (PD) dormancy (*i.e.,* PY+PD) (Baskin & Baskin, 2004). If seeds had PY+PD, the fresh scarified seeds would not have germinated due to the embryo having PD. Mechanical scarification, wet heat (80 °C, 10 min) and alternating wet heat and ice water (five and ten cycles) effectively broke dormancy of *M. pudica* seeds, and the site of water absorption was the hilar region but not the pleurogram.

### Dormancy breaking methods

In a meta-analysis of the most effective methods for breaking PY in seeds of the megagenus *Astragalus* (Fabaceae, subfam. Papilionoideae) by Soltani et al. (2020), mechanical and chemical (immersion in sulphuric acid) scarification were by far the most effective ways to overcome PY. Our research found that mechanical scarification, wet heat (80 °C, 10 min) and alternating wet heat and ice water (five and ten cycles) were effective in breaking PY in seeds of *M. pudica*. Germination of *M. pudica* seeds was >80% after exposure to wet heat (80 °C, 10 min), which was higher than that of seeds exposed to dry heat >80 °C for 10 min (25%). However, the percentage of dead seeds of *M. pudica* was higher for seeds exposed to wet heat than to dry heat, especially at 80 °C for 30 min and 95 °C for 10 min and 30 min. The reason may be that wet heat imposes a more severe effect on seed viability through accelerated ageing than dry heat (Daws et al., 2007; Ku et al., 2015).

Germination (27%) of intact *M. pudica* seeds incubated at 40/30 °C was significantly higher than that at the other four (lower) temperature regimes tested, suggesting that high fluctuating temperature might break dormancy in some seeds of this species. However, germination of non-scarified seeds of *M. foliolosa* (Silveira & Fernandes, 2006) and *M. calodendron* (Dayrell et al., 2015) with PY was 36.0% and 36.5% at a constant temperature of 35 °C, respectively, which was significantly higher than that of seeds incubated at the lower (constant) test temperatures. Thus, it is worth noting that germination (52%) of intact *M. pudica* seeds incubated at 40 °C was higher than that at the fluctuating temperature of 40/30 °C, suggesting that high temperatures play a major role in breaking seed dormancy of *M. pudica* rather than alternating temperature *per se*. It is not likely that tolerance to high temperatures in the habitat (which are much lower than the high oven temperatures that break PY in the laboratory) nor PY per se is the cause of invasiveness of *M. pudica.* PY also is present in noninvasive, rare endemic and species of *Mimosa* that grow in habitats where summer temperatures can be relatively high.

### Seed imbibition site of *M. pudica*

Results of the blocking experiment indicate that the hilar region is the area of water imbibition of *M. pudica* seeds (section *Mimosa*) but that the pleurogram is not. Germination of *M. pudica* was 87% for non-blocked wet-heat treated (nondormant) seeds and 69% for seeds with the pleurogram blocked, while seeds with the whole surface blocked and those with the hilar region blocked germinated to only 4%. SEM analysis suggest that the lens, micropyle and/or hilum is the site(s) of water intake in *M. bimucronata* (section *Batocaulon*) (Geisler et al., 2017). Our SEM results showed that there were obvious cracks and slits in the lens of seeds after they were wet heated at 80 °C for 10 min, which also suggested the lens is a fragile region and likely is a (the) water gap. The hilum and micropyle became wider after *M. pudica* seeds were wet heated at 80 °C for 10 min. Our results agree with those of Geisler et al. (2017), who found that seeds of *M. bimucronata* (in which PY had been broken) with the pleurogram blocked germinated to almost 70%, while those with the hilar region blocked did not germinate.

## Conclusion

In conclusion, seeds of *M. pudica* have PY, and exposure to wet heat at 80 °C for 10 min broke dormancy by creating (an) opening(s) in the hilar region. Thus, in *M. pudica*, the site(s) of water intake was (were) the lens, hilum and/or micropyle. Mechanical scarification, chemical scarification (immersion in sulphuric acid) and wet heat were the most effective treatments breaking PY in seeds of the other 36 species (Table 2). Summer temperatures may break dormancy of *M. pudica* seeds, but neither tolerance to high temperature nor PY is likely to be the cause of invasiveness of *M. pudica.* Considering that our survey included species of *Mimosa* with a wide taxonomic (37 species in four of the five sections of the genus), geographic and habitat representation of the genus, it seems safe to conclude that most (if not all) species in the genus have PY.

##  Supplemental Information

10.7717/peerj.13567/supp-1 Supplemental Information 1 Effect of dormancy-breaking treatments on, and geographical distribution/ecological status of, various taxa of *Mimosa*. +, germination percentage significantly higher than that of control or if statistical test was not done ≥30% of the control; ++, treatment with the highest germination when multiple treatments were given for the same study; ++*, treatment with the highest germination percentage when multiple treatments were given in different studies; -, no significant increase in germination percentage or < 30% of control; yes, imbibition of scarified and non-scarified seeds compared; M, authors said that seeds had physical dormancy. Geographical distribution data are from the Royal Botanical Gardens, Kew, Plants of the World online, 6 August 2021.

10.7717/peerj.13567/supp-2 Supplemental Information 2 Increase in mass (mean % ±se) of mechanically scarified and intact seeds of *M. pudica* after 25 h

10.7717/peerj.13567/supp-3 Supplemental Information 3 Germination percentage of scarified and intact seeds of *M. pudica* at different temperature regimes in light

10.7717/peerj.13567/supp-4 Supplemental Information 4 Effects of alternating cycles of wet heat and ice water on germination percentage (C) and germination rate (1/*t*_50(d)_) (D) of intact seeds of *M. pudica*

10.7717/peerj.13567/supp-5 Supplemental Information 5 Cumulative germination curves for *M. pudica* seeds with hilar region, pleurogram, all of seed surface or nothing (CK) blocked

10.7717/peerj.13567/supp-6 Supplemental Information 6 Effects of dry heat and wet heat treatments on germination at 25/15 °C and viability of intact seeds of *M. pudica* Different lowercase letters indicate significant differences among different temperatures and times under the same treatment (*P* < 0.05). CK is intact seed with no dormancy breaking treatment, *i.e.,* control.

## Additional Information and Declarations

### Data Availability

The following information was supplied regarding data availability: The raw measurements are available in the Supplementary Files.

## References

[ref-1] Aigbokhan EI, Osazuwa-Peters OL, Ilubon KO (2010). Range and distribution of *Mimosa diplotricha* in Nigeria and effects of fire on seed germination. Nigerian Journal of Botany.

[ref-2] Araujo MDS, Andrade GDC (1983). Métodos para superar a dormência tegumentar em sementes de jurema-preta (*Mimosa hostilis* Benth.). Boletim de Pesquisa Florestal, Colombo.

[ref-3] Barneby RC (1991). Sensitivae censitae. A description of the genus Mimosa Linnaeus (Mimosaceae) in the New World. Memoirs of the New York Botanical Garden.

[ref-4] Baskin JM, Baskin CC (2004). A classification system for seed dormancy. Seed Science Research.

[ref-5] Baskin JM, Baskin CC (2014). Seeds: ecology, biogeography, and evolution of dormancy and germination. Second edition.

[ref-6] Bianchetti A (1981). Comparação de tratamentos para superar a dormência de sementes de bracatinga (*Mimosa scabrella* Bentham). Boletim de Pesquisa Florestal, Colombo.

[ref-7] Biondi D, Leal L (2008). Tratamentos pré-germinativos em sementes de *Mimosa strobiliflora* Burkart. Scientia Agraria.

[ref-8] Brito A, Pinto M, Araújo A, Souza V (2014). Superação de dormência em *Mimosa ophthalmocentra* Mart. ex Benth. Enciclopédia Biosfera.

[ref-9] Camargo-Ricalde SL, Dhillion SS, Garcıa-Garcıa V (2004). Phenology, and seed production and germination of seven endemic *Mimosa* species (Fabaceae-Mimosoideae) of the Tehuacán-Cuicatlán Valley, Mexico. Journal of Arid Environments.

[ref-10] Camargo-Ricalde SL, Grether R (1998). Germinación dispersión y establecimiento de plántulas de *Mimosa tenuiflora* (Leguminosae) en México. Revista de Biología Tropical.

[ref-11] Chauhan BS, Johnson DE (2008). Seed germination and seedling emergence of giant sensitiveplant (*Mimosa invisa*). Weed Science.

[ref-12] Chauhan BS, Johnson DE (2009). Germination, emergence, and dormancy of *Mimosa pudica*. Weed Biology and Management.

[ref-13] Chen DL, Zhang R, Baskin CC, Hu XW (2019). Water permeability/impermeability in seeds of 15 species of *Caragana* (Fabaceae). PeerJ.

[ref-14] Corner EJH (1976). The seeds of dicotyledons. Two volumes.

[ref-15] da Rosa FC, Reiniger LRS, Golle DP, Muniz MFB, Curti AR (2012). Overcome dormancy and in vitro germination of seeds of *Mimosa scabrella* Bentham. Semina: Ciências Agrárias.

[ref-16] da Silva ARM, Farias ML, da Silva DLS, Vitoriano JO, de Sousa RC, Alves-Junior C (2017). Using atmospheric plasma to increase wettability, imbibition and germination of physically dormant seeds of *Mimosa caesalpiniafolia*. Colloids and Surfaces B: Biointerfaces.

[ref-17] Daws MI, Kabadajic A, Manger K, Kranner I (2007). Extreme thermo-tolerance in seeds of desert succulents is related to maximum annual temperature. South African Journal of Botany.

[ref-18] Dayrell RLC, De Jesus Gonçalves-Alvim S, Negreiros D, Fernandes GW, Silveira FAO (2015). Environmental control of seed dormancy and germination of *Mimosa calodendron* (Fabaceae): implications for ecological restoration of a highly threatened environment. Brazilian Journal of Botany.

[ref-19] Dillon S, Forcella F (1985). Fluctuating temperatures break seed dormancy of catclaw mimosa (*Mimosa pigra*). Weed Science.

[ref-20] Dos Santos MR, De Oliveira MA, De Oliveira CLLG (2013). Evaluation of methods for germination induction in *Mimosa caesalpiniaefolia* Benth. seeds. Revista Sodebras.

[ref-21] Fowler JAP, Carpanezzi AA (1997). Tratamentos pré-germinativos para sementes de juqueri. EMBRAPA-CNPF. Comunicado Tecnico.

[ref-22] Fowler JAP, Carpanezzi AA (1999). Tratamentos para superação da dormência de sementes de *Mimosa pilulifera* Bentham. EMBRAPA-CNPF. Comunicado Tecnico.

[ref-23] Gama-Arachchige NS, Baskin JM, Geneve RL, Baskin CC (2013). Identification and characterization of ten new water gaps in seeds and fruits with physical dormancy and classification of water-gap complexes. Annals of Botany.

[ref-24] Geisler GE, Pinto TT, Santos M, Paulilo MTS (2017). Seed structures in water uptake, dormancy release, and germination of two tropical forest Fabaceae species with physically dormant seeds. Brazilian Journal of Botany.

[ref-25] Giasson C, Baretta CRDM, Sobral LS, Baldissera R (2019). Dormancy breaking, germination, and production of *Mimosa bimucronata* (DC.) Kuntze seedlings. Cerne.

[ref-26] Giordani L (2008). The role of goats in germination and dispersal of *Mimosa luisana* Brandegee (Leguminosae-Mimosoideae) seeds in Tehuacán-Cuicatlán Valley, Puebla State, Mexico. Master Thesis.

[ref-27] González-Castañeda J, Angoa-Pérez MV, Frías-Hernández JT, Olalde-Portugal V, Flores-Ancira E, Terrones-Rincón TRL, Van Cleemput O, Dendooven L (2004). Germination of seeds of huisache (*Acacia schaffneri*) and catclaw (*Mimosa monancistra*) as affected by sulphuric acid and mechanical scarification and subsequent growth and survival in a greenhouse and field experiment. Seed Science and Technology.

[ref-28] Holm LG, Plucknett DL, Pancho JV, Herberger JP (1977). The world’s worst weeds. Distribution and biology.

[ref-29] Hu XW, Wang Y (2021). The role of the lens in controlling physical dormancy breakdown in three legume species. The XXI International Grassland Congress/VIII International Rangeland Congress, Hohhot, China, June 29 through July 5, 2008. https://uknowledge.uky.edu/igc.

[ref-30] Jayasuriya KG, Wijetunga AS, Baskin JM, Baskin CC (2013). Seed dormancy and storage behaviour in tropical Fabaceae: a study of 100 species from Sri Lanka. Seed Science Research.

[ref-31] Joseph B, George J, Mohan J (2013). Pharmacology and traditional uses of *Mimosa pudica*. International Journal of Pharmaceutical Sciences and Drug Research.

[ref-32] Kopooshian H, Isely D (1966). Seed character relationships in the Leguminosae. Proceedings of the Iowa Academy of Science.

[ref-33] Ku L, Cui X, Cheng F, Guo S, Chen YH (2015). Genetic dissection of seed vigour under artificial ageing conditions using two joined maize recombinant inbred line populations. Plant Breeding.

[ref-34] Leal JV, Alves EU, Bruno RDLA, Pereira W, Alves A, Galindo EA, Alves AU (2008). Épocas de colheita e tratamento pré-germinativos para superação da dormência de sementes *Mimosa caesalpiniifolia* Benth. Revista Árvore.

[ref-35] Leal L, Biondi D (2007). Comportamento germinativo de sementes de *Mimosa dolens* Vell. Ponta Grossa.

[ref-36] Li JH (2018). Study on the suitability of main invasive plant in Hainan and the influencing factors of their population dynamics. Master’s thesis.

[ref-37] Li R, Chen L, Wu Y, Zhang R, Baskin CC, Baskin JM, Hu X (2017). Effects of cultivar and maternal environment on seed quality in *Vicia sativa*. Frontiers in Plant Science.

[ref-38] Liu HL, Abudureheman B, Baskin JM, Baskin CC, Zhang DY (2016). Seed development in the rare cold desert sand dune shrub *Eremosparton songoricum* and a comparison with other papilionoid legumes. Plant Species Biology.

[ref-39] Long Y, Tan DY, Baskin CC, Baskin JM (2012). Seed dormancy and germination characteristics of *Astragalus arpilobus* (Fabaceae, subfamily Papilionoideae), a central Asian desert annual ephemeral. South African Journal of Botany.

[ref-40] Mabberley DJ (2017). Mabberley’s plant-book. A portable dictionary of plants, their classification and uses.

[ref-41] Manning JC, Van Staden J (1987). The systematic significance of testa anatomy in the Leguminosae—an illustrated survey. South African Journal of Botany.

[ref-42] Marambe B, Amarasinghe L, Silva K, Gamage G, Dissanayake S, Seneviratne A, Julien M, Flanagan G, Heard T, Hennecke B, Paynter Q, Wilson C (2004). Distribution, biology and management of *Mimosa pigra* in Sri Lanka. Research and Management of *Mimosa pigra*.

[ref-43] Montaño Arias SA, Camargo-Ricalde SL, Grether R, Díaz-Pontones D (2015). Effect of scarification and temperature on seed germination of two Mexican species of *Mimosa* (Leguminosae-Mimosoideae). Botanical Sciences.

[ref-44] Montaño Arias SA, Camargo-Ricalde SL, Grether R, Díaz-Pontones D (2022). Seed morphology, anatomy and histochemistry in two Mexican species of *Mimosa* (Leguminosae, mimosoid clade). Flora.

[ref-45] Montaño Arias SA, Zavaleta-Mancera HA, Camargo-Ricalde SL, Grether R (2021). Effect of seed age on germination, seedling survival and growth of *Mimosa luisana* (Leguminosae). Trees.

[ref-46] Moreno-Casasola P, Grime JP, Martínez ML (1994). A comparative study of the effects of fluctuations in temperature and moisture supply on hard coat dormancy in seeds of coastal tropical legumes in Mexico. Journal of Tropical Ecology.

[ref-47] Nava-Rodriguez RVT (1974). Estudios sobre ecofisiología de la germinación en tres especies de leguminosas ruderales. Facultad de Ciencias. Tesis de Licenciatura.

[ref-48] Nawa B (2014). Seed germination studies of *Mimosa diplotricha* C. Wright. Indian Forester.

[ref-49] Niroula B, Parajuli D, Jha S (2009). Ecophysiology of *Mimosa pudica* L. at Biratnagar, Eastern Nepal. Our Nature.

[ref-50] Nogueira AC (1996). Superação da dormência de sementes de *Mimosa bimucronata* (DC.) O. Kuntze (maricá). Revista Brasileira de Sementes.

[ref-51] Orozco-Almanza MS, de León-Garcıa LP, Grether R, Garcıa-Moya E (2003). Germination of four species of the genus *Mimosa* (Leguminosae) in a semi-arid zone of Central Mexico. Journal of Arid Environments.

[ref-52] Rana U, Nautiyal AR, Bisht SS (2007). Coat imposed dormancy in *Mimosa himalayana* seeds. Journal of Mountain Research.

[ref-53] Ribeiro MI, Rodrigues GAG, Mathias GL, Silva SM, Corsato JM, Fortes AMT (2020). Effect of seed coat rupture on the germination of *Mimosa flocculosa* Burkart (Leguminosae) seeds. Hoehnea.

[ref-54] Rizzini CT (1976). Influência da temperatura sobre a germinação de diásporos do cerrado. Rodriguésia.

[ref-55] Rodrigues-Junior AG, Baskin CC, Baskin JM, De-Paula OC (2021). The pleurogram, an under-investigated functional trait in seeds. Annals of Botany.

[ref-56] Rodrigues-Junior AG, Mello A, Baskin CC, Baskin JM, Oliveira D, Garcia QS (2019). A function for the pleurogram in physically dormant seeds. Annals of Botany.

[ref-57] Salomão AN (2002). Tropical seed species’ responses to liquid nitrogen exposure. Brazilian Journal of Plant Physiology.

[ref-58] Shibata M, Pavelski LG, Miranda L, de Oliveira LM (2017). Germinação de sementes de *Mimosa flocculosa*. Magistra.

[ref-59] Silva LM, Fernandes GW (2014). Effect of seed storage on germination, seedling growth and survival of *Mimosa foliolosa* (Fabaceae): implications for seed banks and restoration ecology. Tropical Ecology.

[ref-60] Silveira FAO, Fernandes GW (2006). Effect of light, temperature and scarification on the germination of *Mimosa foliolosa* (Leguminosae) seeds. Seed Science & Technology.

[ref-61] Soltani E, Baskin JM, Baskin CC, Benakashani F (2020). A meta-analysis of the effects of treatments used to break dormancy in seeds of the megagenus *Astragalus* (Fabaceae). Seed Science Research.

[ref-62] Sperandio HV, Lopes JC, Matheus MT (2013). Superação de dormência em sementes de *Mimosa setosa* Benth. Comunicata Scientiae.

[ref-63] Walker K (2006). Stratification and scarification techniques to improve the germination rates of herbaceous Mimosa (Mimosa strigillosa). Master’s Thesis.

[ref-64] Wang J, Chen W, Ma R, Baskin CC, Baskin JM, Qi W, Chen X (2016). Role of short-term cold stratification on seed dormancy break and germination of alien species in southeastern China. Plant Ecology.

[ref-65] Yousif MAI, Wang YR, Hu XW (2019). Seed dormancy and dormancy breaking of selected *Acacia* species from Sub-Saharan Africa. Seed Science and Technology.

[ref-66] Zanetti M, Dayrell R, Wardil MV, Damasceno A, Fernandes T, Castilho A, Santos F, Silveira F (2020). Seed functional traits provide support for ecological restoration and ex situ conservation in the threatened Amazon ironstone outcrop flora. Frontiers in Plant Science.

[ref-67] Zirondi HL, Silveira FA, Fidelis A (2019). Fire effects on seed germination: heat shock and smoke on permeable vs impermeable seed coats. Flora.

